# Where to Step? Contributions of Stance Leg Muscle Spindle Afference to Planning of Mediolateral Foot Placement for Balance Control in Young and Old Adults

**DOI:** 10.3389/fphys.2018.01134

**Published:** 2018-08-21

**Authors:** Mina Arvin, Marco J. M. Hoozemans, Mirjam Pijnappels, Jacques Duysens, Sabine M. Verschueren, Jaap H. van Dieën

**Affiliations:** ^1^Department of Human Movement Sciences, Vrije Universiteit Amsterdam, Amsterdam Movement Sciences, Amsterdam, Netherlands; ^2^Department of Movement Sciences, Faculty of Kinesiology and Rehabilitation Sciences, KU Leuven, Leuven, Belgium; ^3^Department of Rehabilitation Sciences, Faculty of Kinesiology and Rehabilitation Sciences, KU Leuven, Leuven, Belgium

**Keywords:** proprioception, muscle spindles, balance, gait, aging, stability

## Abstract

Stable gait requires active control of the mediolateral (ML) kinematics of the body center of mass (CoM) and the base of support (BoS) in relation to each other. Stance leg hip abductor (HA) muscle spindle afference may be used to guide contralateral swing foot placement and adequately position the BoS in relation to the CoM. We studied the role of HA spindle afference in control of ML gait stability in young and older adults by means of muscle vibration. Healthy young (*n* = 12) and older (age > 65 years, *n* = 18) adults walked on a treadmill at their preferred speed. In unperturbed trials, individual linear models using each subject’s body CoM position and velocity at mid-swing as inputs accurately predicted foot placement at the end of the swing phase in the young [mean *R*^2^ = 0.73 (*SD* 0.11)], but less so in the older adults [mean *R*^2^ = 0.60 (*SD* 0.14)]. In vibration trials, HA afference was perturbed either left or right by vibration (90 Hz) in a random selection of 40% of the stance phases. After vibrated stance phases, but not after unvibrated stance phases in the same trials, the foot was placed significantly more inward than predicted by individual models for unperturbed gait. The effect of vibration was stronger in young adults, suggesting that older adults rely less on HA spindle afference. These results show that HA spindle afference in the stance phase of gait contributes to the control of subsequent ML foot placement in relation to the kinematics of the CoM, to stabilize gait in the ML direction and that this pocess is impaired in older adults.

## Introduction

Stable gait, defined as gait that does not lead to falls (Bruijn et al., 2013), requires control of the position of the body center of mass (CoM) relative to the base of support (BoS), formed by those parts of the feet that are in contact with the floor at any point in time. Human bipedal gait has as a disadvantage that a large part of the total body mass is located high above a small BoS. Consequently, small deviations from a vertical orientation result in substantial gravitational moments that accelerate the CoM away from the BoS. The extrapolated CoM, a virtual point based on CoM position and velocity, should fall within the lateral borders of the BoS (Hof et al., 2005). The margin between the border of the BoS and the extrapolated CoM has been coined the margin of stability (Hof et al., 2005). During single-limb support, this margin reaches its minimum value, as the BoS is narrow, while the CoM moves toward the BoS lateral border (Hof et al., 2007a).

Modeling suggests that mediolateral (ML) gait stability requires active, online control (Bauby and Kuo, 2000), which can be achieved by controlling CoM movement through the stance leg (Arvin et al., 2016a), or by controlling the BoS by adjusting ML foot placement with the swing leg (Arvin et al., 2016b). ML foot placement can have substantial effects on CoM acceleration through the moment that the ground reaction force under the foot exerts on the body. With foot placement, large changes in this moment arm can be achieved at relatively low actuation costs, since only the mass of the leg needs to be moved. Consequently, foot placement appears to be the dominant mechanism for maintaining stability of bipedal gait in the ML direction (Hof et al., 2010; Bruijn and van Dieën, 2018).

Studies on balance control in quiet standing have shown that muscle responses to perturbations can be predicted based on delayed feedback of the CoM kinematic state (i.e., position and its derivatives), in cats (Lockhart and Ting, 2007), as well as in humans (Welch and Ting, 2009). It has been suggested that similarly, in gait, the CoM state is estimated to guide future foot placement (Hof et al., 2007b; Wang and Srinivasan, 2014), which is supported by correlations between kinematics of (proxies of) the body CoM during the swing phase and the subsequent foot placement (Hof et al., 2010; Hurt et al., 2010a; Wang and Srinivasan, 2014). Furthermore, external stabilization with springs pulling bilaterally at pelvis level, decreases lateral displacement of the CoM and coincides with decreased step width and step width variability (Donelan et al., 2004; Veneman et al., 2008; Ijmker et al., 2013), This is also the case if trunk kinematics are constrained without any coupling to the external world (Arvin et al., 2016b). Finally, ML mechanical perturbations of gait cause adjustments of foot placement that are proportional to the changes in the CoM velocity induced by the perturbations (Hof et al., 2010; Vlutters et al., 2016). While correlations between CoM state and later foot placement could also result from passive mechanical coupling of the leg to the rest of the body, active, online control of foot placement is strongly supported by evidence that the electromyographic activity of the HA muscles of the swing leg in perturbed and unperturbed gait is associated with CoM kinematics and subsequent ML foot placement (Hof and Duysens, 2013; Rankin et al., 2014).

With age, balance control deteriorates (Sturnieks et al., 2008; Cofre Lizama et al., 2014) and especially impaired ML balance control has been associated with falls in older people (Hilliard et al., 2008). Older adults generally walk with wider steps than young adults (Maki, 1997; Schrager et al., 2008; Arvin et al., 2016a), but at the same time, margins of stability were found to be smaller and more variable in older adults (Arvin et al., 2016a). In addition, Hurt et al. (2010b) reported a less close relationship between CoM kinematics and subsequent foot placement in old than in young adults, suggesting that this active control process may be impaired. However, they used a model of the relationship between CoM kinematics and foot placement based on group data, consequently the lesser model fit in the older adults may also reflect increased between-subject variance in margins of stability.

The evidence for active control of ML gait stability triggers the question how the brain can estimate the CoM kinematic state. Of the multiple sensory modalities that can contribute to estimation of the CoM state, proprioceptive information derived from the hip abductor muscles (HA) of the stance leg seems to play an important role. HA contraction accelerates the CoM toward the contralateral side (Pandy et al., 2010) and hence HA lengthening signaled by muscle spindles would probably be associated with an opposite movement of the CoM. The role of spindle afference can be probed by mechanical vibration, which activates these sensors, causing an illusory perception of lengthening of the vibrated muscle and triggering actions to counteract the associated movement (Goodwin et al., 1972). In standing, vibration of the HA induces lateral body sway (Cofre Lizama et al., 2016). Previous studies showed that continuous muscle vibration of most lower extremity muscles has limited effects during gait (Ivanenko et al., 2000; Verschueren et al., 2003; Courtine et al., 2007), but phasic HA vibration did affect foot placement (Roden-Reynolds et al., 2015). Specifically, vibration during the stance phase caused a more inward placement of the contralateral foot at the end of its swing, while pelvis position relative to the stance foot was unaffected, suggesting unchanged CoM kinematics. This finding would be in line with an adjustment of foot placement in response to a sensory illusion of changed CoM kinematics.

Previous findings on HA muscle spindle afference in relation to control of ML gait stability were obtained in young adults. In quiet standing, older adults tend to relie more on visual and less on proprioceptive information compared to young adults (Eikema et al., 2012b), possibly due to loss of proprioception with aging (Goble et al., 2011). Hence the role of HA spindle afference in planning of ML foot placement for balance control in older adults needs further investigation.

**FIGURE 1:**
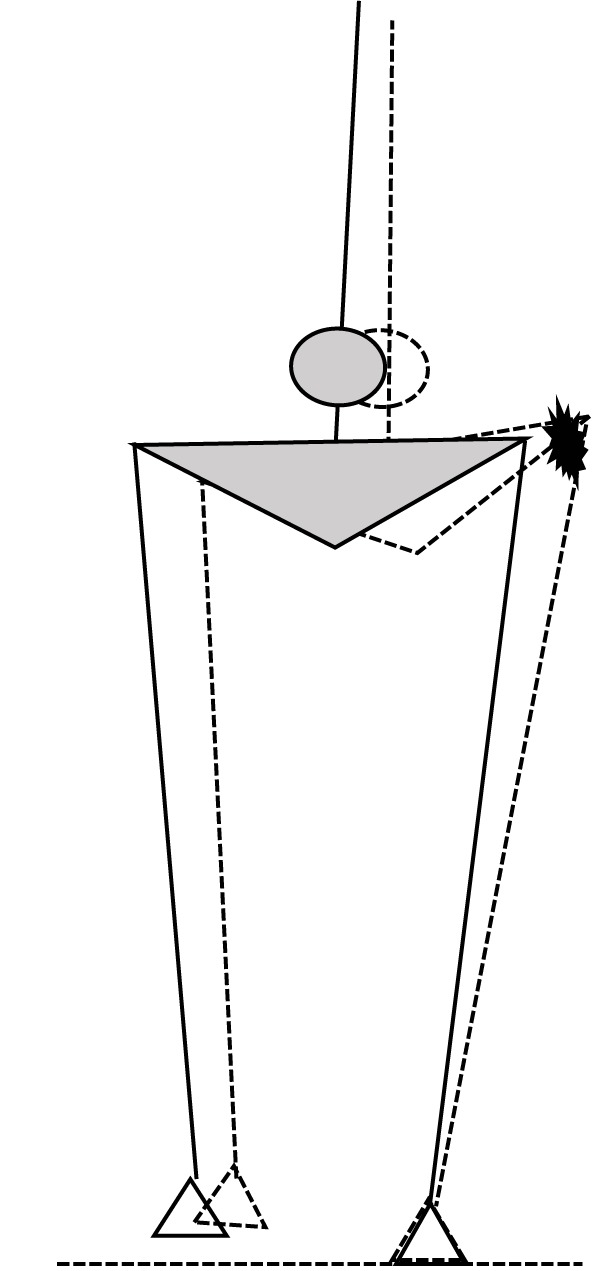
Schematic illustration of the expected illusory effect of stance leg HA muscle vibration, with the solid figure illustrating the actual state in mid swing and the dashed figure illustrating the perceived HA lengthening and associated hip adduction corresponding with an ipsilateral shift of the body CoM (circle), in response to which the contralateral foot will be placed more inward.

In the present study, we combined HA muscle vibration as performed by Roden-Reynolds et al. (2015) with predictive modeling of foot placement based on the CoM state during the preceding stance phase similar to Wang and Srinivasan (2014), to study the coordination between CoM kinematics and foot placement in young and old adults and to assess the role of HA spindle afference in the control of contralateral foot placement. We hypothesized that older adults would show a less close relationship between CoM kinematics during swing and the subsequent foot placement than old adults. In addition, we hypothesized that vibration of the stance leg HA would cause more inward foot placement of the contralateral leg than would be expected based on a predictive model with actual CoM kinematic state as input, due to an illusion of lengthening of the stance leg HA, coinciding with an illusory movement of the CoM toward the ipsilateral side (**Figure 1**). Finally we hypothesized that the vibration effect would be stronger in young adults compared to older adults.

## Materials and Methods

### Participants

Eighteen healthy older adults (10 males and eight females; mean age 70.8 *SD* 6.8 years; height 170.3 *SD* 8.6 cm; weight 80.1 *SD* 8.8 kg) and 12 young adults (six males and six females; mean age 27.3 (*SD* 1.7) years; height 168.0 (*SD* 10.9) cm; weight 60.6 (*SD* 10.5 kg) participated in this study. Prospective participants were excluded if they had a self-reported history of cardiovascular problems, joint disorders, neurological deficits, lower extremity injuries within the last 6 months, postural instability, vestibular or visual problems, and any difficulty with walking without walking aids or with non-stop walking for at least 15 min.

### Ethics Statement

All participants provided written informed consent and the protocol had been approved by the ethical committee of the Department of Human Movement Sciences of the Vrije Universiteit Amsterdam (ECB 2014-22).

### Instrumentation

To apply HA vibration, a pair of custom-made mechanical vibrators was used, which consisted of two encased electromotors (Graphite Brushes S2326.946, Maxon, Sachseln, Switzerland) with an eccentric mass attached to the motor axis, driven in a velocity loop at 90 Hz (4-Q-DC Servo Control LSC 30/2, Maxon, Sachseln, Switzerland). Vibrators were bilaterally placed at 50% of the distance between the iliac crest and the greater trochanter of the femur and were tightly attached with an elastic strap. Custom-made software controlled the vibrators to allow stimulation to be coupled to the kinematics in the gait trials.

Kinematic data were collected using Optotrak (Northern Digital Inc., Waterloo, Canada). Optotrak LED marker clusters were attached with elastic straps and tape on the posterior surface of both heels, shanks, thighs, the sacrum, and the thorax at the midpoint between the scapulas. Marker locations were tracked by two Optotrak camera systems and digitized at 50 samples/s. Ground reaction force data were measured by two force plates embedded in the treadmill (ForceLink, Culemborg, Netherlands) and digitized at 200 samples/s. The force time series were down-sampled to be synchronized with the kinematic data. Kinematic and force data were low-pass filtered with a cut-off frequency of 5 Hz.

### Gait Trials

The participants were asked to walk on the treadmill at their preferred speed (determined according to Arvin et al., 2015) for 3 min without any perturbation (reference trial), followed by a 3-min left or a right HA vibration trial in random order. In these trials, vibration stimuli were delivered unilaterally, randomly in 40% of the stance phases. The real-time position of the Optotrak marker located at the lowest level of the heel cluster was used for online detection of heel strike (HS) and toe-off (TO). The events were estimated to occur when the vertical position of the heel marker crossed the 5 cm line. Consequently, vibration started between ipsilateral HS and contralateral TO and lasted approximately until ipsilateral TO. Participants rested between the two vibration trials if needed.

### Data Analysis

Data were analyzed in MATLAB (The MathWorks, Inc., MA, United States). Before the gait trials, pre-defined anatomical landmarks were digitized in an upright posture, using a probe with six markers. Based on these virtual bony landmarks, anatomical coordinate systems (ACS) were constructed for each segment. The foot ACS was constructed based on the tip of the second toe, calcaneus, and lateral/medial malleolus. The shank ACS was defined by the lateral and medial malleoli and femoral epicondyle. The thigh ACS was based on the lateral and medial femoral epicondyles and the greater trochanter. For the pelvis ACS, the left and right anterior-superior iliac spines, midpoint between posterior-superior iliac spines and the umbilicus were used. The abdomen ASC was based on the umbilicus, the xiphoid and L2, and the thorax was based on the xiphoid, the suprasternal notch, T6 and C7. The mass of each segment was estimated based on segment endpoints (segment lengths) and segment circumference (Zatsiorsky, 2002).

Ground reaction force data were converted to center-of-pressure data, to offline identify the HS and TO events (Roerdink et al., 2008) and to segment the data in strides.

As 40% of the left and right stance phases were randomly vibrated in the vibration trials, preceding or subsequent steps could be perturbed and this might affect outcomes. Therefore, to select non-vibrated steps for analysis, these were defined as steps during which no vibration was applied and for which the preceding and subsequent steps were not vibrated. Similarly, vibrated steps were selected when preceding and subsequent steps were not vibrated. The first 10 strides of each trial were excluded and the rest of the available non-vibrated steps and vibrated steps for the vibration trial as well as the available steps for the reference trial were included in further analyses. Note that we had both left and right steps in the vibration trial. For outcome measures described below, the left steps after right vibration and right steps after left vibration were averaged after adjusting the sign conventions. The same was done for non-vibrated steps.

### Outcome Measures

Mediolateral foot placement was defined based on the heel position trajectory. Body CoM was calculated as a weighted sum over eight segments, after adding the estimated arm and head masses to the thorax segment (Faber et al., 2013). The ML body CoM velocity was calculated as the first derivative of body CoM position time-series. Time-series of ML CoM position and velocity and of ML foot positions were separated in strides and these strides were time-normalized. CoM state at the 80% time point in the gait cycles (mid-swing) and foot positions at HS 100% of the gait cycles were referenced to the ipsilateral foot positions at 0% of the gait cycles. For each participant and each foot, a predictive foot placement model was formed based on these data. The model linked the variance in ML foot placement at heel strike to the variance in ML CoM position and velocity during the preceding swing phase (c.f. Wang and Srinivasan, 2014) as:

$$\text{FP=β1}\cdot {\text{CoM}}_{\text{mid-swing}}+\text{β2}\cdot {\text{VCoM}}_{\text{mid-swing}}+\text{ε}$$

To avoid an intercept in the model all variables were referenced to their respective averages. Separate models were developed for left and right foot placement using the CoM state at 80% (mid-swing) of the gait cycle. Goodness of fit was determined as the variance in actual foot placement accounted for by the predicted foot placement (*R*^2^-values). The results section reports models based on mid-swing predictions. To verify the robustness of these models and of age effects on goodness of fit, alternative models predicting foot placement from CoM states at 85%, 90%, 95%, and 100% (HS) of the gait cycle were developed (**Supplementary Material**).

Subsequently, we applied the models developed from the reference trial to the vibration trial, analyzing the vibrated and non-vibrated steps separately. The difference between model prediction and actual ML foot placement was determined as the primary outcome for vibration effects and will be referred to as foot placement adjustment. These differences were averaged within subjects over steps and over left and right legs after adjustment of the sign convention. If the model accurately predicts foot placement in the vibration trials, foot placement adjustments for the non-vibrated steps should not be significantly different from zero, while in vibrated steps, adjustments in foot placement would result in more inward placement of the foot than predicted by the model, due to a mismatch between the actual CoM state and the disturbed CoM state estimate, as outlined in the introduction section (**Figure 1**). To assess recovery from effects of vibration, we also assessed foot placement at the subsequent, contra-lateral step using the same methodology.

### Statistics

For all statistical tests, the assumption of normality was checked by the Shapiro–Wilks test and no violation of this assumption was found. The *R*^2^-values for the goodness of fit predicted to actual foot placements in the reference trials were compared between age-groups, using an independent samples *t*-test. Repeated measures analysis of variance with two factors (vibration condition [non-vibrated step, vibrated step] × age [young, old]) was used to compare the differences of within-subject averaged CoM position and velocity at mid-swing between vibrated and non-vibrated steps in the vibration trials. Repeated measures analysis of variance with two factors (vibration condition [non-vibrated step, vibrated step, subsequent step] × age [young, old]) was used to compare foot placement adjustments between non-vibrated and vibrated steps and between groups. *Post hoc*, *t*-tests were done to compare foot placement adjustments between age groups and between vibration conditions, with Bonferroni corrections where applicable. In addition, to compare foot placements in the vibration trials to those in the reference trials, one-sample *t*-tests were used to assess whether adjustments in foot placement were different from zero. For all analyses, *p*-values below 0.05 were considered significant.

## Results

### Descriptive Data

The average preferred walking speeds were 1.02 (*SD* 0.2) m/s in the young group and 0.7 (*SD* 0.2) m/s in the old group and were significantly different between groups (*p < 0.001*). The reference trials contained on average 156 steps in the young group and 187 steps in the old group. After selection of the non-vibrated and vibrated steps, the vibration trials comprised on average 40 (*SD* 7) non-vibrated steps and 41 (*SD* 6) vibrated steps across the young and old groups. The average duration of the vibration stimuli was 0.41 (*SD* 0.04) s.

### Foot Placement Prediction

**FIGURE 2:**
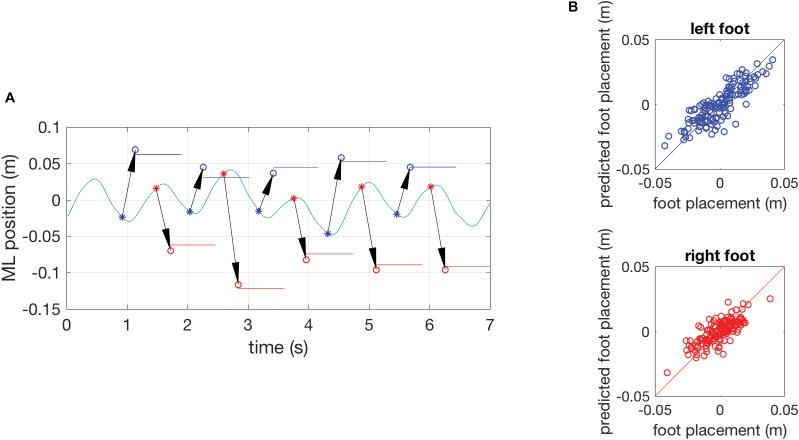
**(A)** Results of a single subject showing the time series of the mediolateral position of the COM during gait in part of an unperturbed reference trial with the actual foot-placement positions and their duration indicated as colored horizontal lines and model predictions of foot-placement based on the COM position and velocity at preceding mid-swing as circles (o). The arrows represent the prediction and connect the COM state at mid-swing (^∗^, the predictor variable) to the predicted foot-placement at the instant of foot contact. Data for the left foot are presented in blue, data for the right foot in red. **(B)** Quality of the model prediction illustrated by scatter plots of actual and predicted foot-placements of the complete reference trial. The blue circles in the top graph represent left foot placement and red circles in the bottom graph represent right foot placement. The diagonal lines are identity lines.

**Figure 2** illustrates the model inputs and predictions for a short episode of gait in the reference trial of a representative participant and shows scatter plots of all actual and predicted foot placements in the same subject. Across all subjects, the model with CoM position and velocity at the preceding mid-swing as predictors accurately predicted ML foot placement, as reflected in high variance accounted for, with mean *R*^2^-values of 0.73 (*SD* 0.11) in the young and 0.60 (*SD* 0.14) in the old group. As hypothesized, mean *R*^2^-values were significantly different between the age groups (*p < 0.05*). This effect was consistent regardless of which phase in the gait cycle was used as a predictor of foot placement (**Supplementary Material**).

### Vibration Effects

There were no significant differences in mean CoM position at mid-swing between reference and vibration trials, nor between non-vibrated and vibrated steps in the vibration trials, nor were there any significant differences between the young and old group (**Table 1**). COM velocity at mid-swing was not significantly different between trials, but was significantly lower in the older group (**Table 1**), presumably due to the lower gait speed in this group.

**Table 1:** Descriptives of the mean (*SD*) CoM ML kinematics at mid-swing of the non-vibrated and vibrated steps in the young and old group and *p*-values for repeated measures ANOVA.

				Vibration	Age	Vibration × age
			
		Non-vibrated steps	Vibrated steps	*P*-value	*P*-value	*P*-value
CoM ML position (cm)	Young adults	2.3 (0.8)	2.1 (0.8)	0.709	0.716	0.268
	Older adults	2.3 (0.6)	2.3 (0.6)			
CoM ML velocity (cm/s)	Young adults	9.3 (2.2)	9.0 (2.5)	0.237	**0.001**	0.286
	Older adults	5.6 (1.1)	5.6 (1.3)			

*Bold values indicate significant effects (*p* < 0.05)*.

**FIGURE 3:**
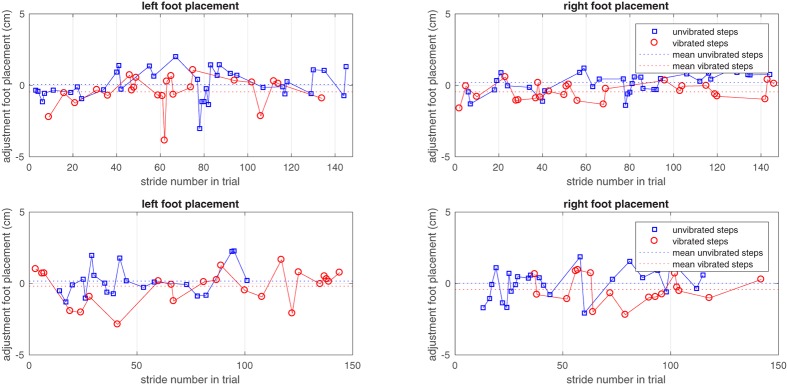
The adjustment of left and right foot placements in non-vibrated and vibrated steps in one young and one older subject over the vibration trial. Only steps taken into consideration in the analysis are represented as symbols. Zero adjustment refers to the foot placement predicted by the model based on the unperturbed reference trial.

**Figure 3** illustrates the adjustments in foot placement in the non-vibrated and vibrated steps. The figure shows systematic deviations from zero, indicating the effects of vibration compared to the reference trial. In addition, the figure shows the systematic differences in foot placement adjustments between vibrated and non-vibrated steps. No trend-wise variation of foot placement adjustment over time is observable, suggesting that there was no habituation to vibration. This was confirmed by regression analyses of foot placement adjustments in vibrated steps against stride number in the trial, yielding coefficients of correlation that tended toward zero (young adults *r* = -0.06, *SD* = 0.19; older adults *r* = -0.01, *SD* = 0.11).

Foot placement adjustments were significantly different between non-vibrated and vibrated steps (*F*_2,56_ = 75.9, *p* < 0.001). They were not affected by age (*F*_1,28_ = 0.2, *p* = 0.666), but the interaction between vibration condition and age was significant (*F*_2,56_ = 7.0, *p* = 0.002).

**FIGURE 4:**
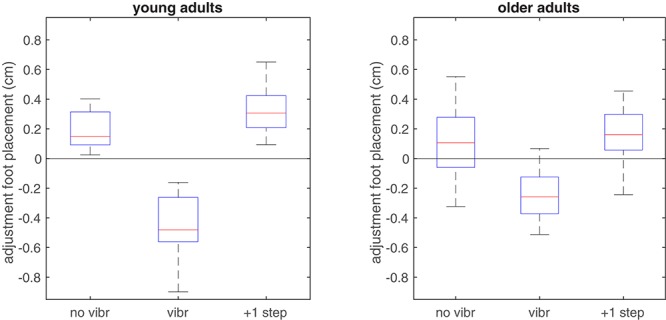
Boxplot of adjustments in foot placement relative to the predictive model derived from the reference trials, when applied to non-vibrated and vibrated steps in the vibration trials. In vibrated steps, adjustments relative to predicted foot placement were significantly more toward inward in the young group, in line with the expected illusory inward movement of the CoM associated with lengthening of the HA muscles. Zero adjustment refers to the foot placement predicted by the model based on the unperturbed reference trial.

As hypothesized the mean foot placement adjustment in vibrated steps indicated an inward adjustment in response to the vibratory stimulus (**Figure 4**). This was significant in both groups (*p* < 0.001 in both groups), but the interaction effect indicated larger foot placement adjustments after vibration in the young compared to the older group (*p* = 0.007). For the non-vibrated steps, foot placement adjustments indicated a more outward foot placement in the vibration trials than in the reference trials (*p* < 0.001 in young adults and *p* = 0.061 in older adults). The difference between age groups was not significant (*p* = 0.247). The second step after the vibration stimulus, foot placement was again more outward and even somewhat more so than in the non-vibrated steps in young adults (*p* = 0.030 and *p* = 0.426 and in young and older adults, respectively). The difference between age groups for this second step after the vibration stimulus was significant (*p* = 0.019).

## Discussion

We investigated the role of HA muscle spindle afference in the control of ML gait stability in terms of the coordination between the ML CoM kinematic state at mid-swing and subsequent ML foot placement in young and old adults. Foot placement was more tightly coordinated with CoM kinematics in the preceding swing phase in young than in older adults. When HA afference was experimentally perturbed by unilateral muscle vibration during the stance phase, this resulted in more inward contralateral foot placement at the end of the swing phase, indicating the importance of muscle spindle afferent information in regulating the ML BoS with respect to the body CoM state during walking. This effect was stronger in the young than in older adults, suggesting that HA spindle afference contributes less to the control of ML gait stability in older than in young adults.

### Foot Placement Prediction

Our results support previous findings indicating that ML foot placement is regulated based on the CoM position and velocity in the preceding swing phase. Similar to previous work (Wang and Srinivasan, 2014), we found that over 70% of the ML foot placement variance in young adult is predictable based on the ML CoM position and velocity at mid-swing. In previous studies, foot placement was predicted based on position and acceleration (Hurt et al., 2010a) or position and velocity data (Wang and Srinivasan, 2014; Vlutters et al., 2016) in the preceding swing phase. Our results confirm that ML CoM kinematic state plays an important role in regulating stable gait. Furthermore, previous studies used the trunk CoM (Hurt et al., 2010a) or pelvis CoM (Wang and Srinivasan, 2014) instead of whole-body CoM to predict foot placement. We also checked these alternative predictors and found similar predictive models, yet with a slightly better predictive value when using the total body CoM instead of trunk or pelvis CoM and no substantial improvement in predictive value when adding acceleration as a predictor.

In older adults, the variance in foot placement accounted for by the kinematic state of the CoM during the middle of the preceding swing phase was lower than in young adults. This suggests less tight coordination between foot placement and CoM kinematics in the older adults. However, it was recently shown that the coupling between CoM kinematics and foot placement increases with gait speed in young adults (Stimpson et al., 2018), which might indicate that the difference between age groups was mediated by the lower gait speed in the older adults. Since speed and age were highly correlated in our data set, using speed as a covariate in the analysis was not feasible. Redoing the statistical analysis using dichotomized speed as a factor instead of dichotomized age showed similar effects on the *R*^2^-values of the foot placement prediction model as age did. However, Stimpson et al. (2018) argued that the lower correlation between COM state at mid-swing at lower speeds in young adults did not represent less tight control but rather an earlier control of foot placement at higher speeds, since speed effects on *R*^2^-values vanished when CoM state later in the gait cycle was used as a predictor of foot placement. In contrast, differences in variance of foot placement accounted for by CoM kinematic state between age groups stayed constant when using CoM states later in the gait cycle in the present study (**Supplementary Material**). In addition, our previous finding of a greater variability of ML margins of stability in older than in young adults (Arvin et al., 2016a) would be in line with less tight control of foot placement in older adults, whereas variability of ML margins of stability was found to increase with gait speed in young adults (Stimpson et al., 2018). Finally, since the two groups were walking at their preferred speed, rendering conditions representative of their normal gait, we do believe that the finding of a less tight coupling between foot placement would be relevant even if it would be influenced by the fact that older adults walk slower.

### Vibration Effects

The effects of vibration observed here support the idea that afferent feedback is used to actively control the relationship between the ML, CoM state, and ML foot placement during gait (Bruijn and van Dieën, 2018) and add that HA spindle afference during the stance phase of gait is used in this control process. We expected that HA vibration in the stance phase would result in a perception of lengthening of these muscles coinciding with an illusion of inward movement (toward the vibrated stance leg). No differences in CoM kinematic state at mid-swing between vibrated and non-vibrated steps were found. This indicates that stance HA vibration did not cause a tonic vibration reflex with subsequent shortening of the vibrated muscles, since this would result in increased outward acceleration of the CoM toward the non-vibrated leg. This finding supports the idea that a movement illusion rather than an actual change in CoM kinematics led to the foot placement adjustment. Not adjusting the CoM kinematics, but compensating for these by adjusting subsequent swing foot placement may be preferable in view of the lower actuation torques required and the lower energy demand (Kuo, 1999; Donelan et al., 2004; Wert et al., 2010; Kubinski et al., 2015).

While a systematic inward placement of the foot after vibration was found, foot placement in non-vibrated steps was more outward compared to the reference trials. In addition, foot placement in the second step after vibration tended to be even more outward. These findings may reflect a more cautious gait pattern in the vibration trials compared to the reference trials, especially right after vibration, likely due to uncertainty created by the conflict between illusory proprioceptive information and veridical visual and vestibular information. These findings were more pronounced in the young than in the older adults, which is in line with a reduced weighting of proprioceptive information in the older adults. Also, this further emphasizes our findings on the immediate effects of vibration; even though generally subjects walked with wider steps in the vibration trial, right after vibration they stepped more inward, compared to the non-vibrated steps but also compared to the reference trial.

The possibility that vibration in previous steps influenced our results was ruled out by excluding steps that were preceded by steps with vibration from analysis. However, the vibratory stimulus could continue to have effects in subsequent steps beyond the first one. It has been shown that the state of the CoM in the step preceding the one that is considered here has no predictive value for foot placement (Wang and Srinivasan, 2014). As such, apart from the general effect of walking with wider steps, sustained effects of the illusory sensory information induced by vibration should not carry over to subsequent steps. However, any mechanical perturbation induced by the stimulus (i.e., the step too far inward) could have a longer lasting effect. Mechanically this would require a more inward foot placement again on the subsequent step. Therefore, we compared foot placement adjustments among non-vibrated steps, vibrated steps and subsequent steps. The results showed that subsequent steps were placed more outward compared to the reference trials and the vibrated steps, similar to the non-vibrated steps. This suggests that except for the first step after vibration, the effect of vibration was non-specific, in line with non-significant effects of vibration in subsequent steps reported by Roden-Reynolds et al. (2015). Finally, it cannot be completely ruled out that vibration has non-specific effects not mediated by muscle spindle afference; the vibration of any muscle might evoke a protective response. However, one might expect individuals to move away from an unexpected stimulus rather than step toward it, hence the opposite and predicted effect in the vibrated steps seems at odds with such an explanation.

In agreement with previous studies (Sorensen et al., 2002; Glasser et al., 2015; Roden-Reynolds et al., 2015), the effects of HA muscle vibration in gait were small. Combination of veridical information derived from vision, the vestibular system, and other sub-modalities of the proprioceptive system may have limited the magnitude of responses. In spite of the limited magnitude, the consistent and significant effects of HA muscle vibration indicate that this source of sensory information plays a role in control of ML gait stability.

The present study also revealed that older adults were less responsive to stance phase HA vibration than young adults. This suggests that they relies less on proprioception for control of ML gait stability than young adults, in line with previous findings on control of quiet standing (Eikema et al., 2012a). Subcutaneous fat may affect the transmission of the vibration stimulus to the muscle and body mass and body mass index of our older participants were higher than that of the young participants. Consequently, results may have been confounded by differences in subcutaneous fat. Age and BMI were highly correlated, hence using BMI as a covariate in the analysis was not feasible. Instead, we repeated the repeated measures ANOVA with dichotomized BMI (with 25 kg/m^2^ as cut-off). The interaction effect between vibration and BMI was not significant (*p* = 0.073), indicating that age was a stronger predictor of differential effects of vibration than BMI. Body mass may also affect foot placement adjustments mechanically. Higher leg mass as well as limited muscle strength in the elderly may have reduced the magnitudes of kinematic responses measured. This may have contributed to the effects found, although one might expect that feedback gains would be adjusted to deal with efficacy of the effector system. Another potential confounding factor in the comparison of vibration effects between age groups, is the difference in gait speed between these groups. The amount of time available for adjusting foot placement to the vibration stimulus would be shorter at higher stride frequencies and hence likely at higher speeds. Also, leg angular momentum would be higher at higher gait speeds, hence adjusting the foot path might require more effort. These effects would bias results toward smaller effects in young adults, whereas we found the opposite.

## Conclusion

We found more tight coordination of ML foot placement in young compared to older adults. In addition, we found that HA muscle spindle afference plays a role in the control of ML foot placement relative to the CoM kinematic state in the preceding swing phase, but more so in young than in older adults.

## Author Contributions

MA and JvD designed the study and analyzed the data. MA collected the data. MA, MH, MP, JD, SV, and JvD interpreted the results and wrote and edited the manuscript. All authors have read and agreed with the submitted version.

## Conflict of Interest Statement

The authors declare that the research was conducted in the absence of any commercial or financial relationships that could be construed as a potential conflict of interest.
