# Supplementary material for: Where to Step? Contributions of Stance Leg Muscle Spindle Afference to Planning of Mediolateral Foot Placement for Balance Control in Young and Old Adults
Source: Front Physiol. 2018 Aug 21;9:1134. doi: 10.3389/fphys.2018.01134 (PMC6110888; doi:10.3389/fphys.2018.01134)
Supplement: Supplementary file 1 [file Table_1.DOCX]

Supplementary material

**Comparison of goodness of fit of models predicting foot placement based on CoM states**

**Figure S.1** Boxplots of R^2^-values of models predicting foot placement based on CoM states at different phase of the gait cycle.

Repeated measures analysis of variance with two factors (age [young×old] x phase of the gait cycle [80%, 85%, 90%, 95% and 100%]).

| phase | age | age x phase |
| --- | --- | --- |
| F_4,108_ = 39.2 | F_1,28_ = 12.2 | F_4,108_ = 0.3 |
| p < 0.001 | p = 0.002 | p = 0.881 |
